# Transactions

**Published:** 1880-06

**Authors:** 


### TRANSACTIONS.

The Transactions of the nineteenth annual session of the American Dental Association, held at Niagara Falls, Aug. 5th, 1879, is just from the press. It is a volume of 134 pages, in which the minutes, reports and discussions are well presented. The publication committee and the printers have done their work well. The transactions of this body should be in the hands of every member of the profession who has any ambition to keep pace with its progress. Only in the meetings and by the doings of our Associations can the real progress and growth of the profession be properly estimated. And as matter of future reference these volumes will be invaluable. The reports and discussions should be closely studied; in them will be found some good professional pabulum.

Only a small edition has been issued; this is unfortunate, but circumstances are sometimes inflexible; the publication committee were no doubt made aware of that fact. Every member of the Association who stands well in the eyes—or on the books—of our treasurer is entitled to the volume, and any extra numbers can be had by such parties at $1 per volume, and others may obtain them at $1.25 per volume.

They can be had by application to the secretary of the Association, Dr. Geo. H. Cushing, No. 34 Monroe St., Chicago.

The procurement of the volume will be more certain by early application.

